# Supplementary material for: Continually recruited naïve T cells contribute to the follicular helper and regulatory T cell pools in germinal centers
Source: Nat Commun. 2023 Oct 31;14:6944. doi: 10.1038/s41467-023-41880-9 (PMC10618265; doi:10.1038/s41467-023-41880-9)
Supplement: Supplementary file 1 — Supplementary Information [file 41467_2023_41880_MOESM1_ESM.pdf]

Supplementary Information for:

Continually recruited naïve T cells contribute to the follicular helper and regulatory T cell pools in germinal centers.

Julia Merkenschlager[1*], Riza-Maria Berz[1], Victor Ramos[1], Maximilian Uhlig[1], Andrew J. MacLean[1], Carla R. Nowosad[2], Thiago Y. Oliveira[1], Michel C. Nussenzweig[1,3,]

[1]Laboratory of Molecular Immunology, The Rockefeller University, New York, NY 10065, USA

[2]Translational Immunology Center, New York University Grossman School of Medicine, New York, NY, 10016, USA

[3]Howard Hughes Medical Institute, The Rockefeller University, New York, NY 10065, USA

* Correspondence to: jmerkensch@rockefeller.edu

Supplementary data Inventory

Supplementary Data Figures 1-8

- Supplementary data. Figure 1. Profiling of the *Sell*Cre ROSAtdT (CD62L) reporter mice.
- Supplementary data Figure 2. Novel T_{FH} T cell clonotypes are detected between day 7 and day 21 post immunization.
- Supplementary data Figure 3. Progressive differentiation of naïve tdTomato labelled cells.
- Supplementary data Figure 4. Naïve T cells invade GC reactions.
- Supplementary data Figure 5. Newly differentiated T_{FH} are oligoclonal and clonally expanded.
- Supplementary data Figure 6. Naïve cells that enter the GC reaction late are enriched in $T_{FR.}$
- Supplementary Figure 7. T_{FR} can differentiate from naïve conventional CD4 T cells and naïve tTregs.
- Supplementary Figure 8. Antigen dependent T_{FR} development.

Supplementary Table 1

- Details of antibodies used in the study.

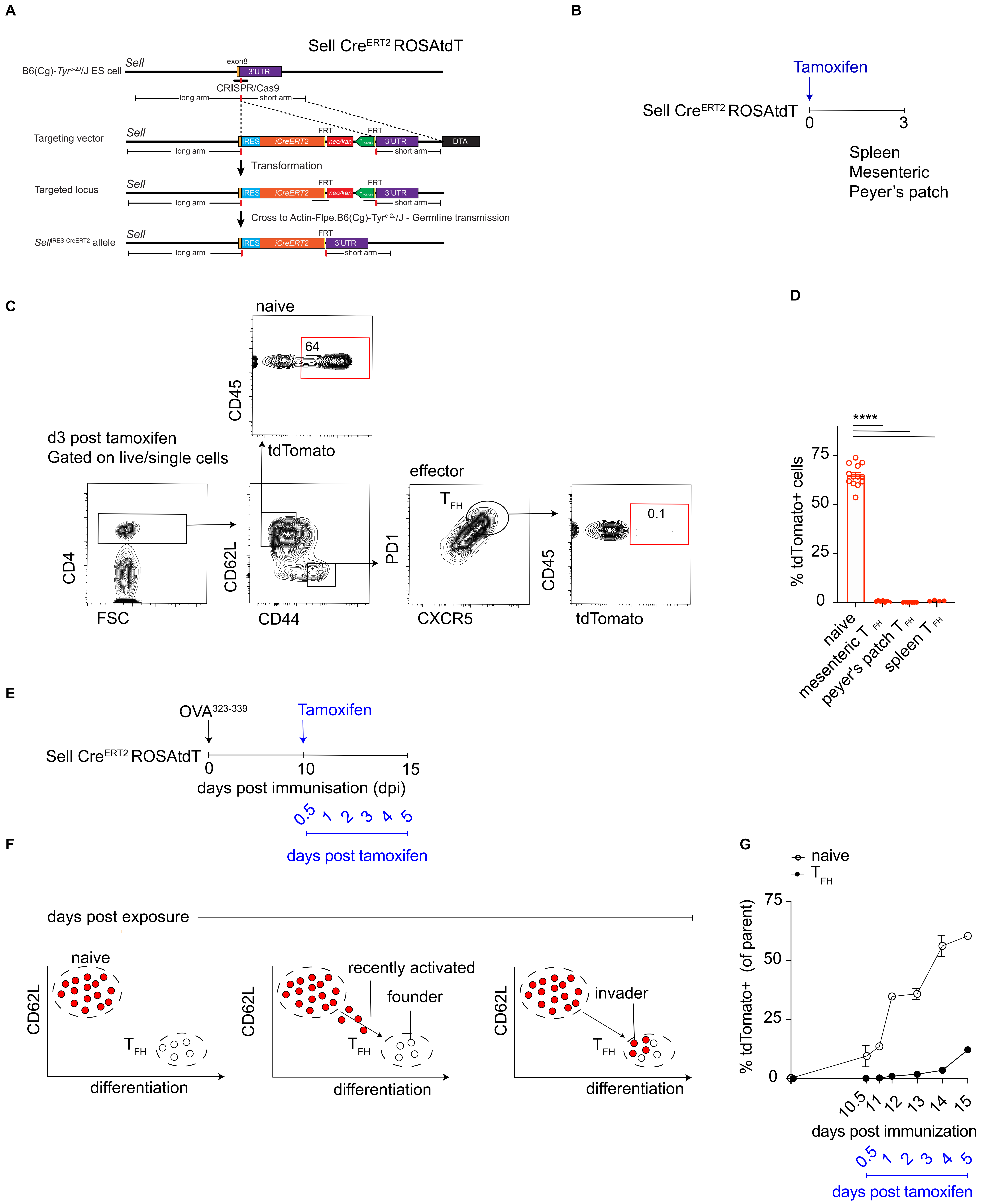

Supplementary data. Figure 1. Profiling of the SellCre ROSAtdT (CD62L) reporter mice. (A) Targeting strategy and the configuration of the SellIRES-CreERT2 allele. The mice were produced at Rockefeller University and crossed to ROSA tdTomatoloxP/loxP to generate SellCre ROSAtdT (CD62L) reporter mice. **(B)** Schematic representation of the experimental setup used in (C and D). **(C)** Representative flow cytometry plots profiling tdTomato expression in naive or T_{FH} cells assayed in unimmunized mice three days after tamoxifen exposure. **(D)** Bar graph depicts the frequency of tdTomato+ cells (Y axis) in naïve or T_{FH} cells from mesenteric, Peyer's patch lymph nodes or in spleen of unimmunized mice three days after tamoxifen exposure. p-values<0.0001 as calculated by unpaired Student's t test (two tailed). n = 4-13 per group and data presented are the mean. ±SEM. **(E)** Schematic and **(F)** diagrammatic representations of the experimental setup and labelling strategies used to visualize naive T cell invasion into GC reactions in (G). **(G)** Graph shows the mean contribution of tdTomato+ cells to naïve of T_{FH} cells when tamoxifen was administered at day 10 post tamoxifen and cells followed thereafter. Y axis shows the mean values of tdTomato+ cells among T_{FH} (filled) or naïve (open) T cells. X axis is the interval days post immunization and days post tamoxifen exposure assayed. n = 3-9 per group and data presented are the mean. ±SD.

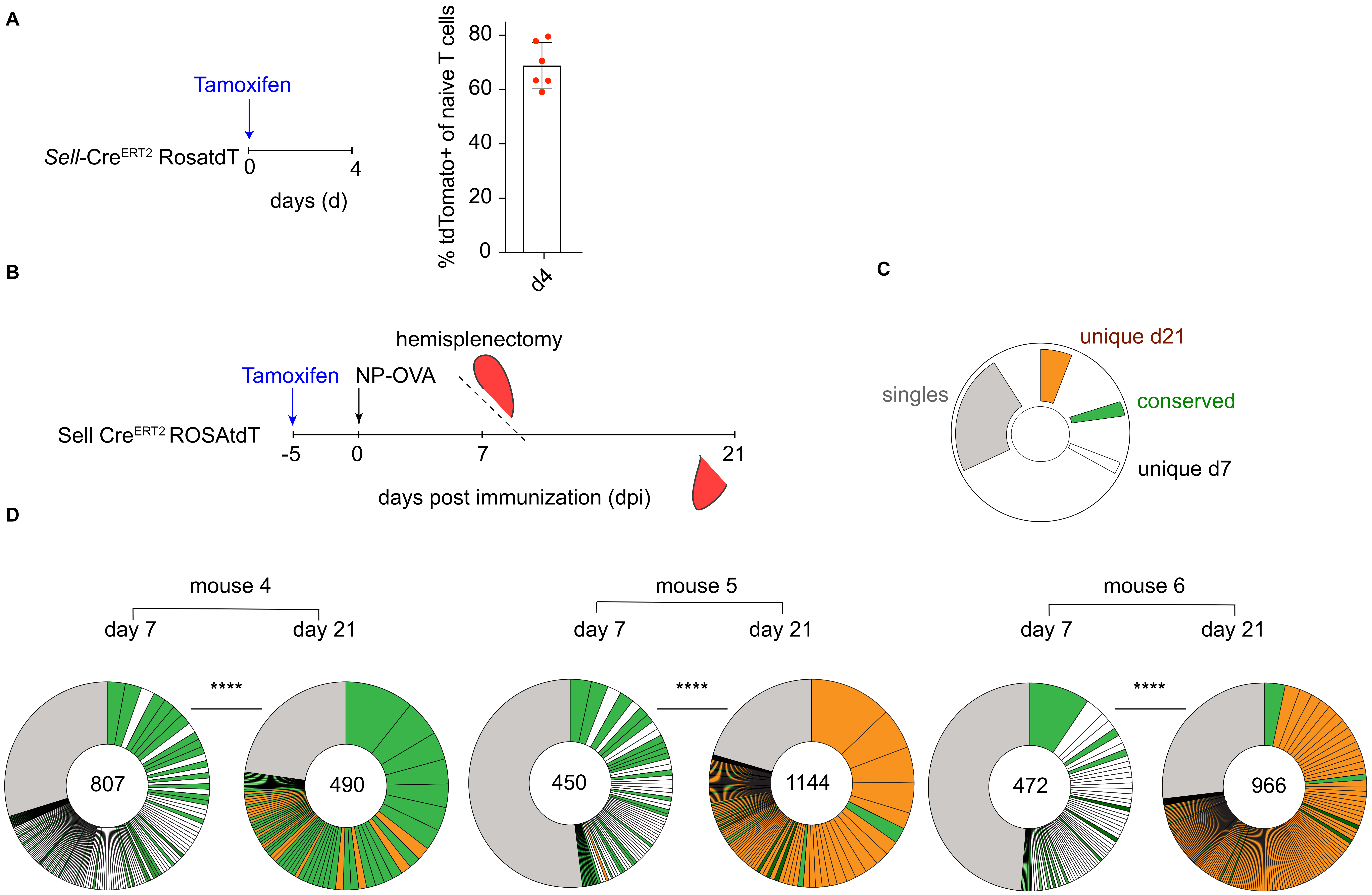

Supplementary data Figure 2. Novel T_{FH} T cell clonotypes are detected between day 7 and day 21 post immunization.
(A) (Left) Schematic representation of the experimental setup in used. (Right) Bar graph depicts the frequency of tdTomato+ cells (Y axis) in naïve T cells four days after tamoxifen exposure (X axis). *n* = 6 and data presented are the mean. ±SD. **(B)** (Left) Schematic representation of the experimental setup in (C and D). **(C)** Color coded indexing for the clonal behaviors between d7 and d21 post immunization in (D). Conserved TCR clonotypes are represented in green, and those found only on d7 in white, novel clones appearing only on d21 in orange and singles in grey. **(D)** Pie charts show clones of T_{FH} cells at the indicated timepoints. Segments are proportional to the representation of each clone. Numbers inside the pie charts indicate the number of TCR sequences. The data discussed have been deposited in the NCBI Gene Expression Omnibus are accessible through GEO series accession number: GSE240730.

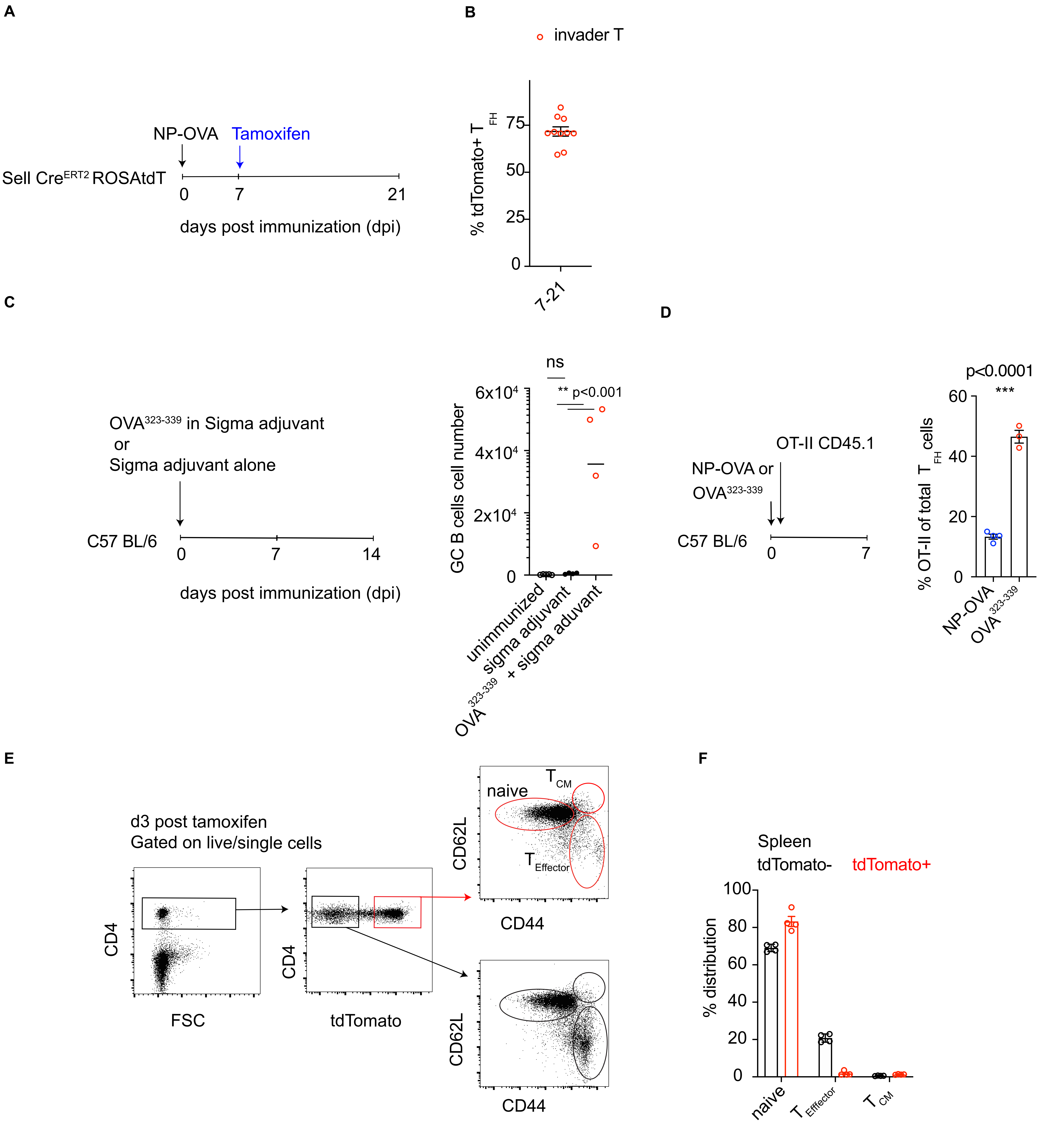

Supplementary data Figure 3. Progressive differentiation of naïve tdTomato labelled cells.
(A) Schematic and of the experimental setup used in B. **(B)** Bar graph to show the contribution of new invading T cells between days 7-21 post immunization. Y axis is the frequency of tdTomato+ cells in T_{FH} cells normalized to labeling in the naïve compartment. X axis is the interval between tamoxifen exposure and assay. n = 10 and data presented are the mean. ±SEM. **(C)** Schematic representation of the experimental setup used in (right). (Right) Graph depicting the total number of GC B cells (Y axis) in the popliteal lymph nodes of unimmunized mice or 14 days following administration of $OVA^{323-339}$ delivered in sigma adjuvant or just Sigma adjuvant alone respectively (X axis). * p-values=0.01 as calculated ANOVA with multiple comparison. *n* = 4 and data presented are the mean. ±SD. **(D)** Schematic representation of the experimental setup used in (right). (Right) Graph depicting the frequency of OT-II cells among total T_{FH} compartment (Y axis) during a diverse or a narrowed response to NP-OVA or $OVA^{323-339}$ respectively (X axis).** p<0.0001 by student's t t-test (two tailed). n = 4 and data presented are the mean. ±SD **(E)** Representative flow cytometry plots profiling naïve, T effector and central memory compartments in tdTomato+ and tdTomato- compartment from the spleens of unimmunized mice three days after tamoxifen exposure. **(F)** Bar graph depicts the frequency of naïve, T central memory cells (TCM) and T effector and T effector memory (TEM) in tdTomato positive and tdTomato negative compartments. *n* = 4 and data presented are the mean. ±SD.

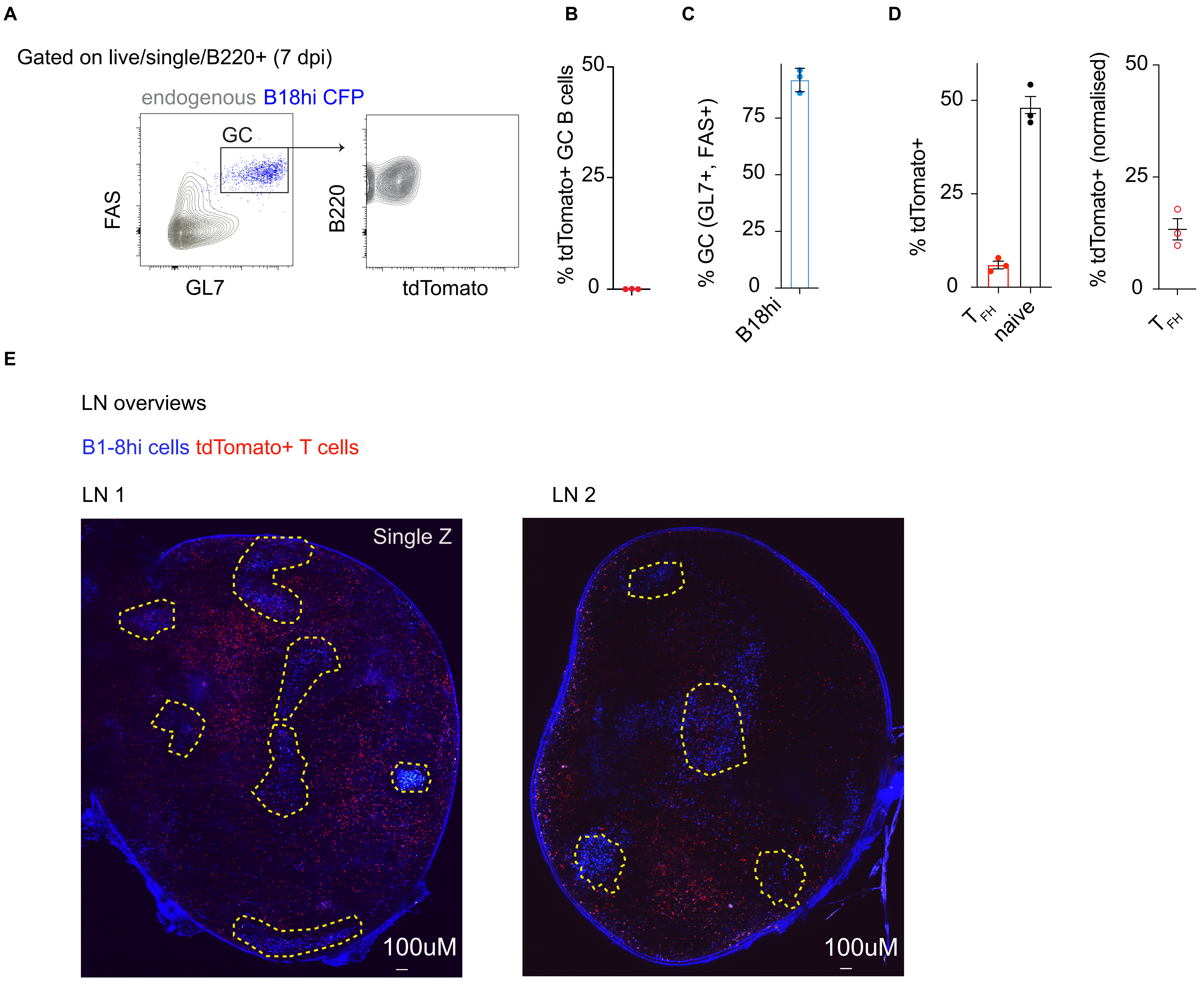

Supplementary data Figure 4. Naïve T cells invade GC reactions.
(A) Representative flow cytometry plots showing the absence of tdTomato labelling in GC B cells quantified in (B). **(B)** Plots showing the percentage of GC B cells (B220^{positive}, CD38^{negative}, CD95^{positive}, GL7^{positive}) that express tdTomato. *n* = 4 and data presented are the mean. ±SD **(C)** Bar graphs plots the percentage of B18hi B cells that differentiated into GC B cells on day 17 post immunization. *n* = 4 and data presented are the mean. ±SD **(D)** Bar graphs plots the percentage of tdTomato^{+} cells among T_{FH} cells or naïve T cells (left) and the normalized percentage of tdTomato^{+} T_{FH} cells (right) on day 17. *n* = 4 per conditions and data presented are the mean. ±SD **(E)** Multi-photon (single Z stack) images that show lymph node overviews to visualize GCs within in popliteal lymph nodes, as defined by the fluorescent-ly labelled B18hi B cells (blue). Data presented are from two separate mice. Individual GCs are marked with dashed yellow lines.

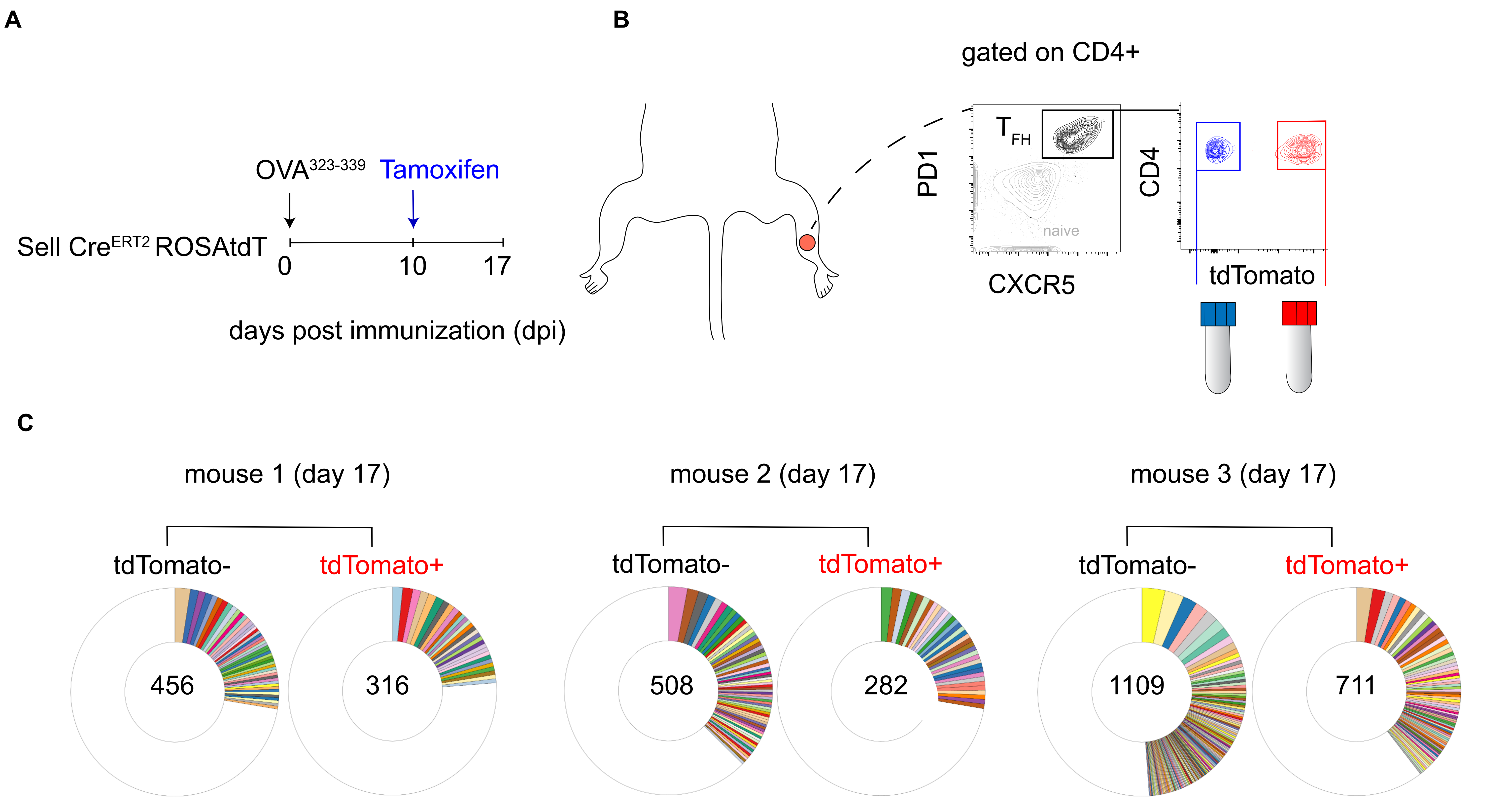

Supplementary data Figure 5. Newly differentiated T_{FH} are oligoclonal and clonally expanded.
(A) Schematic representation of the experimental strategy used in (B and C). **(B)** Representative flow cytometry plots showing the cell sorting strategy used to isolate tdTomato$^+$ and tdTomato$^-$ T_{FH} cells. **(C)** Pie charts show singlets in white or clones in colored slices at the indicated timepoints. Segments are proportional to the representation of each clone. Numbers indicate the number of TCR sequences. The data discussed have been deposited in the NCBI Gene Expression Omnibus are accessible through GEO series accession number: GSE240730.

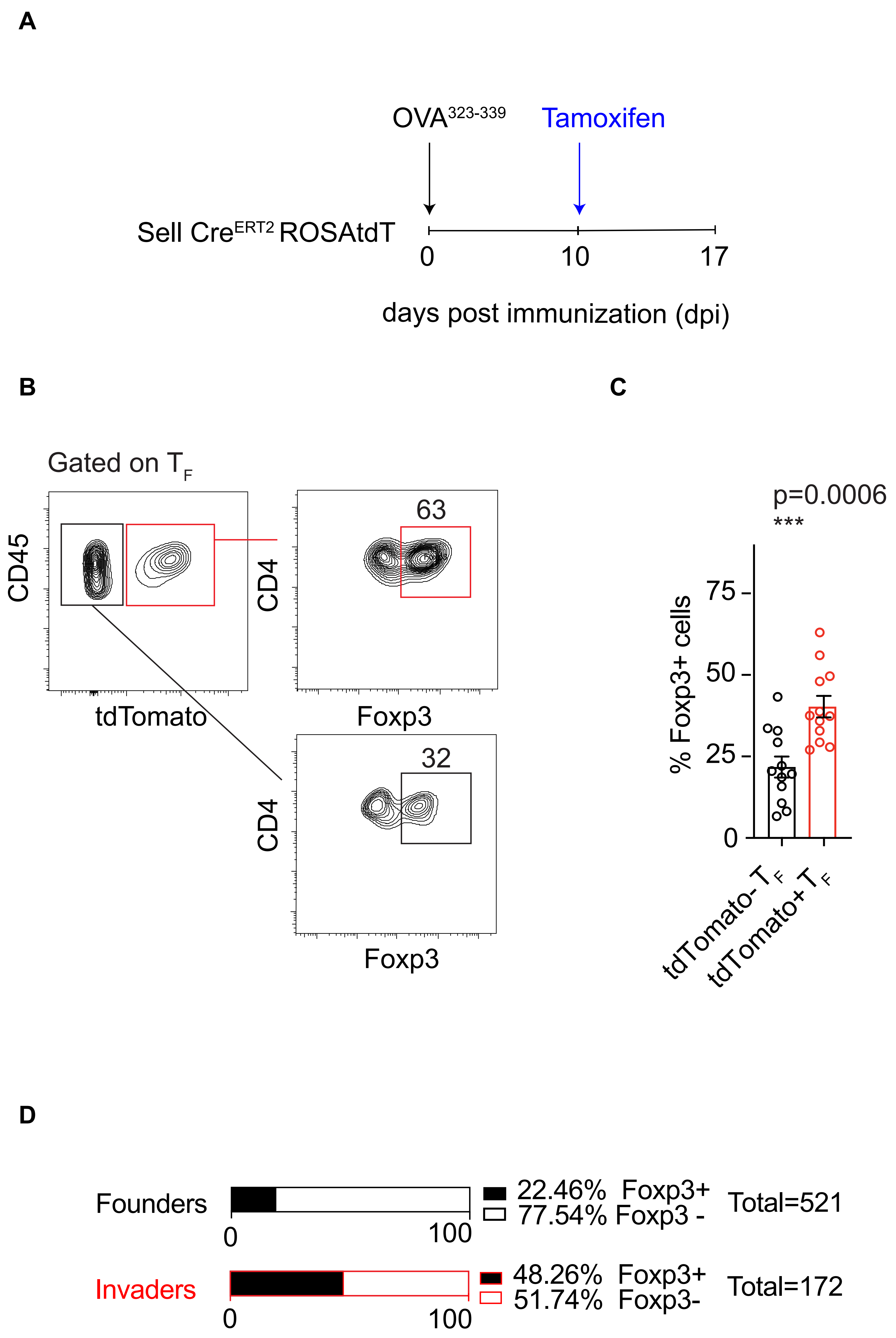

Supplementary data Figure 6. Naïve cells that enter the GC reaction late are enriched in T_{FR}.
(A) Schematic representation of the experimental strategy used in (B and C). **(B)** Representative flow cytometry plots comparing the frequency of cells expressing Foxp3 protein between invader and founder cells, 17 days post immunization. **(C)** Rightmost bar graph compares the percentage of $Foxp3^{+}$ cells on the Y axis in T_F cells between populations defined on the X axis. ***p-values=0.006 by unpaired Student's t test (two tailed). *n* = 12 and data presented are the mean ±SEM.**(D)** Stacked bar charts depicting the frequency of cells that had detectable levels of Foxp3 transcripts in unique founders or unique invaders populations.

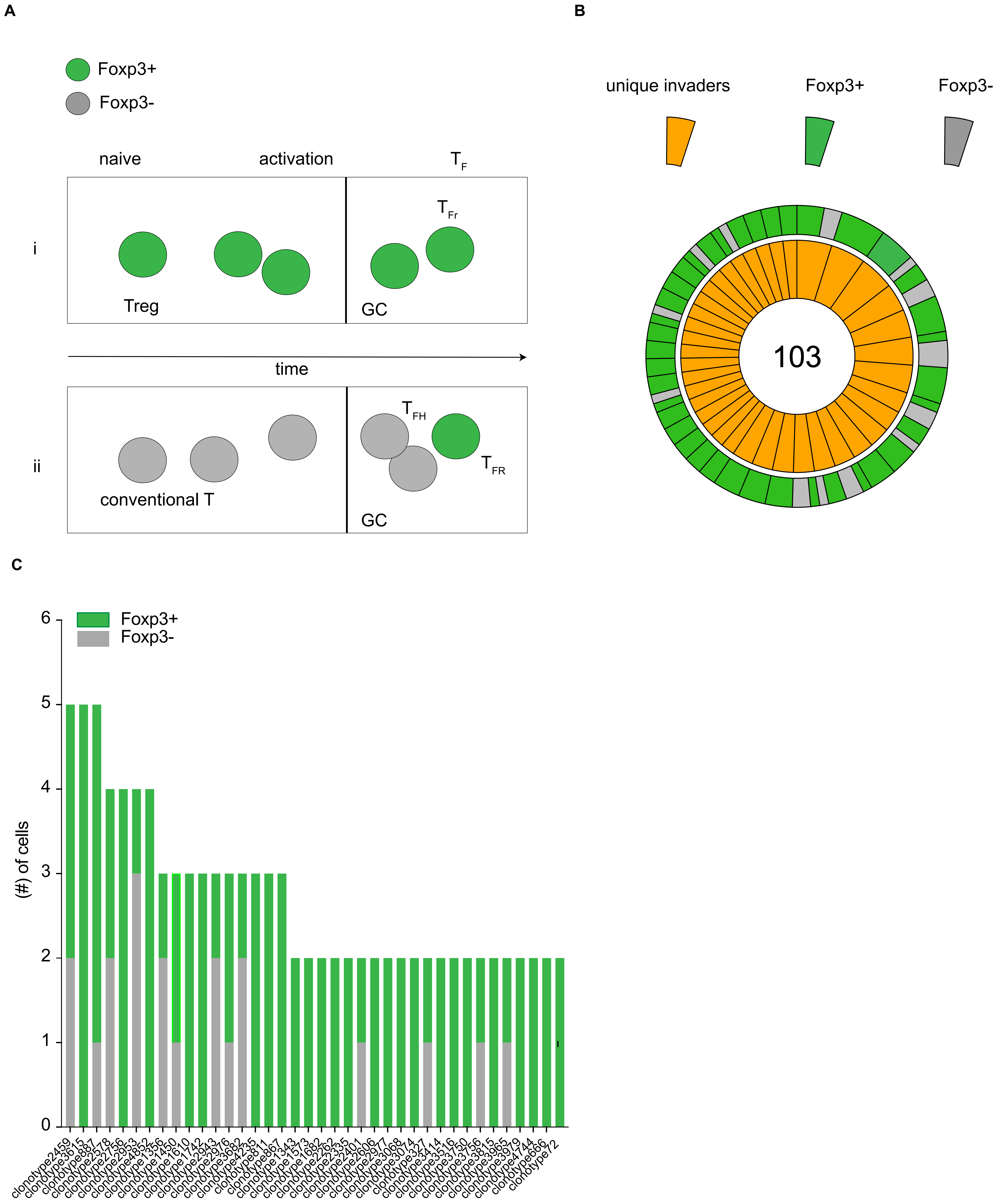

Supplementary data Figure 7. T_{FR} can differentiate from naïve conventional CD4 T cells and naïve tTregs.
(A) Schematic representation of two possible models for T_{FR} differentiation *i)* T_{FR} cells originate from a thymic Treg precursor, resulting in homogenous expression of Foxp3 among all clonal members. *ii)* T_{FR}s originate from a conventional naïve CD4 T cell which later turns on Foxp3 expressors during differentiation, resulting in a "mixed" collection of Foxp3^{+} and Foxp3^{-} clones. **(B)** Pie charts show expanded unique invader clones in which at least one cell within the family expresses Foxp3 transcript (orange). Inner orange segments are proportional to the representation of each expanded clone. Outer segments are the proportion of Foxp3^{+} (green) or Foxp3^{-} cell (grey) within each of the clonal families indicated. Numbers inside the pie charts indicate the number of cells represented. **(C)** The bar graph compares the percentage of Foxp3+ (green) cells and Foxp3- (grey) cells (X axis) with each expanded unique invader clonotypes (Y axis).

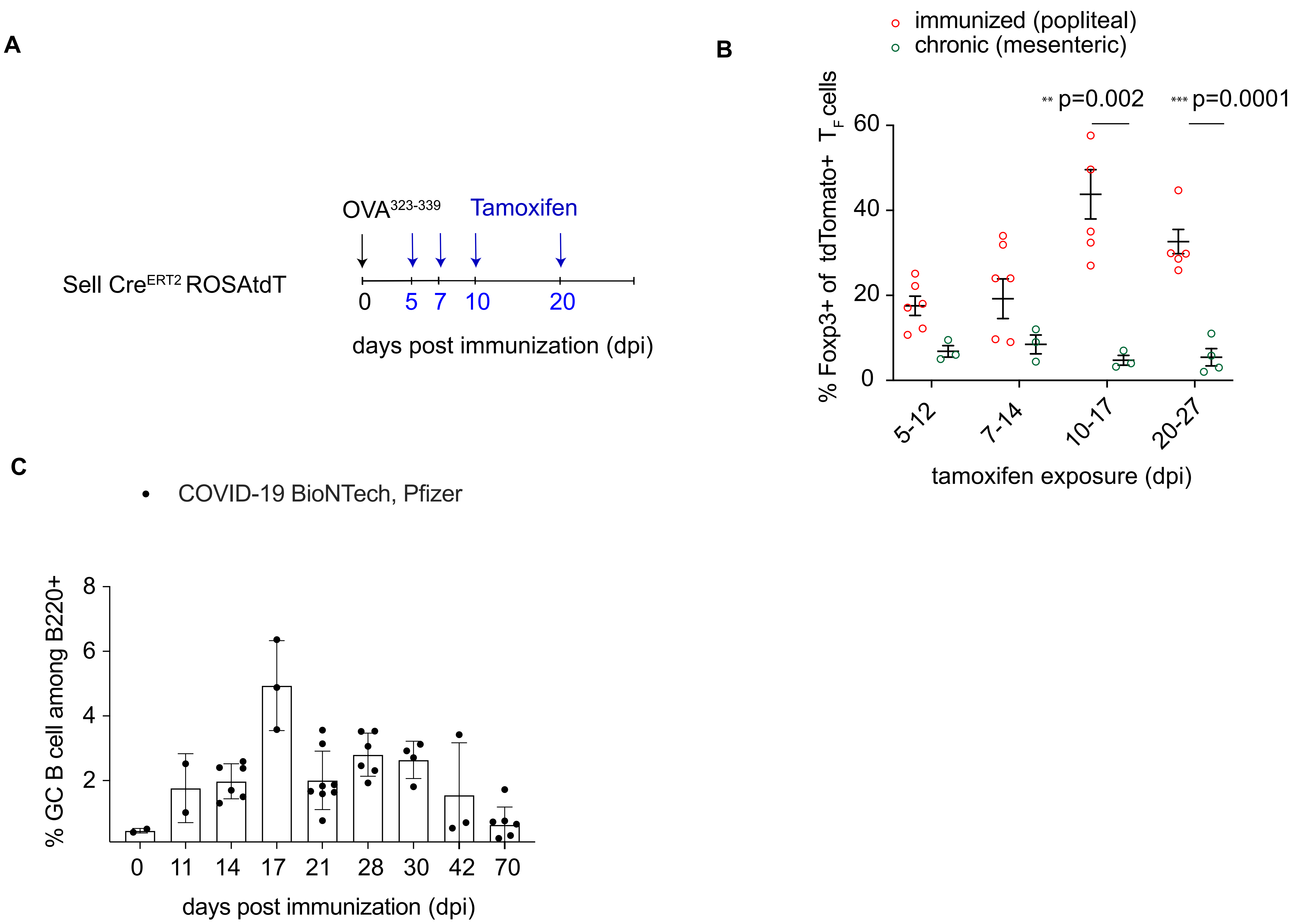

Supplementary data Figure 8. Antigen dependent T_{FR} development.
(A) Schematic representation of the experimental setup used in (B). **(B)** Bar graph shows the frequency of Foxp3⁺ cells among total T_F cells in invaders at the indicated timepoints, in popliteal lymph nodes (red) or in mesenteric lymph nodes (green). * p-value = 0.017, ** p-value = 0.0026, ***p-value =0.0001, calculated by Student's *t* test (two tailed). *n*= 3-6 per group and data presented are the mean ±SEM. **(C)** Bar graph compares the percentage of GC (B220^{positive}, GL7^{positive}, FAS^{positive}) cells among B cells (Y axis) in the days following COVID-19 BioNTech (Pfizer) immunization. *n*= 2-9 per group and data presented are the mean ±SD.
.

Supplementary Table 1

**Antibody** (description/clone/cat)	**dilutions**
D16/CD32 (mouse BD fc), CD16/CD32, Clone 2.4G2, Cat 553142, Lot 9060742,	1/200
BD anti-mouse Foxp3 APC (ebioscience) Clone FJK-16s, Cat 17-5773-82	1/200
anti-mouse foxp3 FITC, eBioscience, clone FJK-16s, Cat 11-5773-82	1/200
anti-mouse Bcl6 PE, Clone K112 91, Cat 561522, Lot 8233984, BD	1/100
FITC anti-human/mouse Bcl-6 Antibody, Cat 358513, Biolegend	1/200
anti-mouse CD4, Clone RM4-5, Cat 100516, Lot B277608, Biolegend	1/200
anti-mouse CD4 APC, Cat 100516, Lot B277608, Biolegend	1/200
anti-mouse CD4, Clone RM4-5, Cat 100516, Lot B277608, Biolegend	1/200
anti-mouse CD4 BUV395; Clone GKl.5, Cat 563790, Lot 9275330, BD	1/200
anti-mouse CD4 PE, Clone GKl.5, Cat 100408, Lot B266388, Biolegend	1/200
anti-mouse CD62L PECY7, Clone MEL-14, Cat 104418, Lot B269976, Biolegend	1/200
anti-mouse CD44, Clone IM7, Cat 103027, Biolegend	1/200
anti-mouse CD44 APC, Clone IM7, Cat 563058, Lot B265921, BD	1/200
anti-mouse CD44 APC, Clone IM7, Cat 563058, Lot B265921, BD	1/200
Biolegend anti-CD44 Fitc, Clone IM7, Cat 11-0441-82, eBioscience	1/200
anti-mouse/human CD44 APC/Cyyanine 7, Cat 103027, Lot B352758	1/200
anti-mouse/human CD44 Brilliant Violet 421m, Clone IM7, Cat 103040, Lot B273304,	1/200
anti-mouse CD185 (CXCR5) bv 421, Cat 145512, Lot B357018, Biolegend	1/200
Biotin Rat anti-mouse CD185 (CXCR5), Cat 145510, Lot B21465, BD	1/200
anti-mouse PDl- BV711, Clone 29F.1Al2, Cat 135231, Lot B298663, Biolegend	1/200
anti-mouse PDl PE, Clone J43, Cat 551892, Lot 7086579, BD Bioscience	1/200
anti-mouse CD279 APC, Clone 29F.1Al2, Cat 109112, Lot B248540, Biolegend	1/200
anti mouse VB 5.1 T cell receptor Clone: MR9-4 Cat 553190	1/200
anti mouse VB 5.1 T cell receptor APC Clone: MR9-4 Cat 139506 Lot B3163556	1/200
anti-mouse CD45.l PE/Cyanine7, Clone A20, Cat 110729, Biolegend	1/200
anti-mouse CD45.1 BV421M, clone A20, Lot B376745, Biolegend.	1/200
anti-mouse CD45.1 FITC, Clone A20, cat 35-0453-U025, Lot C0453120821352, TONBO bioscience	1/200
anti-CD45.2 Mouse Monoclonal Antibody PE, Clone 104, Cat 109808, Lot B271929,	1/200
anti-mouse CD45.2, Clone 104, Cat 109808, Lot B271929, eBioscience	1/200
anti-mouse CD45.2, BV421, Clone 104, Cat 109832, Lot B357158, Biolegend	1/200
anti-mouse CD45.2 APC-Cyanine, clone 104, Cat 109824, Lot B335012, Biolegend	1/200
anti-mouse CD45.2, APC Clone 104, Cat 109814, Lot B338570, Biolegend	1/200
anti mouse CD45R/B220, Cat 563793, Lot 3135095, BD	1/200
anti-mouse CD38, Clone 90/CD38, Cat 553764, BD Bioscience	1/200
anti-mouse CD38, Clone 90/CD38, Cat 102719, Lot B371397, Biolegend	1/200
anti-mouse GL7 PB, Clone GL7, Cat 144614, Lot B306510, Biolegend	1/200
anti-mouse GL7 FITC, Clone GL7, Cat 144603, Biolegend	1/200
anti-mouse Pecy7 CD95, Clone Jo2, Cat 557653, lot 2145378, BD	1/200
anti-NK-1.1 Mouse Monoclonal Antibody PE, Clone PK136, Cat 557391, Lot 65616,	1/200
anti-mouse/human PE CD45R/B220 Antibody, Clone RA3-6B2, Cat 103208, Biolegend	1/200
anti-mouse CD86 APC, Clone GL-1, Cat 4332810, Biolegend	1/200
TotalSeqm-C0301 anti-mouse Hashtag 1 Antibody 155861, Biolegend	1ug
TotalSeqm-C0302 anti-mouse Hashtag 2 Antibody 155863, Biolegend	1ug
TotalSeqm-c0303 anti-mouse Hashtag 3 Antibody 155865,Biolegend	1ug
TotalSeqm-co304 anti-mouse Hashtag 4 Antibody 155867,Biolegend	1ug
TotalSeqm-c0305 anti-mouse Hashtag 5 Antibody, Biolegend	1ug
TotalSeqm-C0306 anti-mouse Hashtag 6 Antibody , Biolegend	1ug
TotalSeqm-C0307 anti-mouse Hashtag 7 Antibody, Biolegend	1ug
TotalSeqm-C0308 anti-mouse Hashtag 8 Antibody ,Biolegend	1ug
TotalSeqm-co309 anti-mouse Hashtag 9 Antibody,Biolegend	1ug
TotalSeqm-C0310 anti-mouse Hashtag 10 Antibody, Biolegend	1ug
