# Supplementary material for: Continually recruited naïve T cells contribute to the follicular helper and regulatory T cell pools in germinal centers
Source: Nat Commun. 2023 Oct 31;14:6944. doi: 10.1038/s41467-023-41880-9 (PMC10618265; doi:10.1038/s41467-023-41880-9)
Supplement: Supplementary file 3 — Reporting Summary [file 41467_2023_41880_MOESM3_ESM.pdf]

nature portfolio

Corresponding author(s): Julia Merkenschlager

Last updated by author(s): 17 August 2023

Reporting Summary

Nature Portfolio wishes to improve the reproducibility of the work that we publish. This form provides structure for consistency and transparency in reporting. For further information on Nature Portfolio policies, see our Editorial Policies and the Editorial Policy Checklist.

Statistics

For all statistical analyses, confirm that the following items are present in the figure legend, table legend, main text, or Methods section.

n/a	Confirmed	
☐	☒	The exact sample size (*n*) for each experimental group/condition, given as a discrete number and unit of measurement
☐	☒	A statement on whether measurements were taken from distinct samples or whether the same sample was measured repeatedly
☐	☒	The statistical test(s) used AND whether they are one- or two-sided<br>*Only common tests should be described solely by name; describe more complex techniques in the Methods section.*
☐	☒	A description of all covariates tested
☐	☒	A description of any assumptions or corrections, such as tests of normality and adjustment for multiple comparisons
☐	☒	A full description of the statistical parameters including central tendency (e.g. means) or other basic estimates (e.g. regression coefficient) AND variation (e.g. standard deviation) or associated estimates of uncertainty (e.g. confidence intervals)
☐	☒	For null hypothesis testing, the test statistic (e.g. *F*, *t*, *r*) with confidence intervals, effect sizes, degrees of freedom and *P* value noted<br>*Give P values as exact values whenever suitable.*
☐	☒	For Bayesian analysis, information on the choice of priors and Markov chain Monte Carlo settings
☐	☒	For hierarchical and complex designs, identification of the appropriate level for tests and full reporting of outcomes
☒	☐	Estimates of effect sizes (e.g. Cohen's *d*, Pearson's *r*), indicating how they were calculated

Our web collection on statistics for biologists contains articles on many of the points above.

Software and code

Policy information about availability of computer code

Data collection	Flow cytometry data was collected using FACDIVA version 8.0.2.
Data analysis	MacVector was used for sequence analysis. Graph Prism 9 was used for data analysis and for graph generation. In addition, We used cellranger (v3.0.2) from 10X Genomics for single-cell UMI quantification and TCR clonotype assembly. We used Seurat (v3.1.2) an R package to analyze single cell RNA-seq data and to identify differentially expressed genes; graphs were created using R language. For bulk-RNA seq analysis we used kallisto (v.0.46) to map sequence reads to Mus musculus transcriptome (GRCm38/ Ensembl release 99). Kallisto TPM values were converted to absolute counts using tximport (v1.12.3) R package and DESeq2 (v.1.24.0) .

For manuscripts utilizing custom algorithms or software that are central to the research but not yet described in published literature, software must be made available to editors and reviewers. We strongly encourage code deposition in a community repository (e.g. GitHub). See the Nature Portfolio guidelines for submitting code & software for further information.

Data

Policy information about availability of data

All manuscripts must include a data availability statement. This statement should provide the following information, where applicable:

- Accession codes, unique identifiers, or web links for publicly available datasets
- A description of any restrictions on data availability
- For clinical datasets or third party data, please ensure that the statement adheres to our policy

The authors declare that all data supporting the findings of this study are available within the article and its supplementary

file or from the corresponding author upon reasonable request. The data discussed in this publication have been deposited Gene Expression Omnibus are accessible through GEO series accession number: GSE147182 and GSE240730.

Human research participants

Policy information about studies involving human research participants and Sex and Gender in Research.

Reporting on sex and gender	N/A
Population characteristics	N/A
Recruitment	N/A
Ethics oversight	N/A

Note that full information on the approval of the study protocol must also be provided in the manuscript.

Field-specific reporting

Please select the one below that is the best fit for your research. If you are not sure, read the appropriate sections before making your selection.

[x] Life sciences   [ ] Behavioural & social sciences   [ ] Ecological, evolutionary & environmental sciences

For a reference copy of the document with all sections, see nature.com/documents/nr-reporting-summary-flat.pdf

Life sciences study design

All studies must disclose on these points even when the disclosure is negative.

Sample size	Groups of 3-20 mice were used in immunizations. Sample size was determined based on the common standard in the field. n>3 mice/group were used and all the animal experiments were repeated 2-3 times. The number of independent samples used in the experiment are reported in the figure legends.
Data exclusions	We did not exclude any samples. Age and sex matched were used in all experiments. Reported in figure legends.
Replication	Each experiment was performed 2-3 times. A total of 6 mice were used to track clonal evolution longitudionally in the same individuals previosuly published (Merkenschlager, J. Nature 2021). A total of 4 mice were used to compare the composition of the fate mappped and non fate mapped cells in day 17 post immunization. Littermates were used as controls, wherever possible. Appropriate controls are used in each experiment.
Randomization	Litter mate controls were used for in house strain. Otherwise, C57BL/6 wild type mice were purchased from The Jackson and divided into sex matched and age matched groups
Blinding	Mice were homogenous in sex and age prior to grouping. Investigators were not blinded in this study

Reporting for specific materials, systems and methods

We require information from authors about some types of materials, experimental systems and methods used in many studies. Here, indicate whether each material, system or method listed is relevant to your study. If you are not sure if a list item applies to your research, read the appropriate section before selecting a response.

Materials & experimental systems

n/a	Involved in the study	
[ ]	[x]	Antibodies
[x]	[ ]	Eukaryotic cell lines
[x]	[ ]	Palaeontology and archaeology
[ ]	[x]	Animals and other organisms
[x]	[ ]	Clinical data
[x]	[ ]	Dual use research of concern

Methods

n/a	Involved in the study	
[x]	[ ]	ChIP-seq
[ ]	[x]	Flow cytometry
[x]	[ ]	MRI-based neuroimaging

Antibodies

Antibodies used	

The updated manuscript includes a complete table detailing all the antibodies used:

Antibody and dilutions

D16/CD32 (mouse BD fc), CD16/CD32, Clone 2.4G2, Cat 553142, Lot 9060742, 1/200
BD anti-mouse Foxp3 APC (ebioscience) Clone FJK-16s, Cat 17-5773-82 1/200
anti-mouse foxp3 FITC, eBioscience, clone FJK-16s, Cat 11-5773-82 1/200
anti-mouse Bcl6 PE, Clone K112 91, Cat 561522, Lot 8233984, BD 1/100
FITC anti-human/mouse Bcl-6 Antibody, Cat 358513, Biolegend 1/200
anti-mouse CD4, Clone RM4-5, Cat 100516, Lot B277608, Biolegend 1/200
anti-mouse CD4 APC, Cat 100516, Lot B277608, Biolegend 1/200
anti-mouse CD4, Clone RM4-5, Cat 100516, Lot B277608, Biolegend 1/200
anti-mouse CD4 BUV395; Clone GKl.5, Cat 563790, Lot 9275330, BD 1/200
anti-mouse CD4 PE, Clone GKl.5, Cat 100408, Lot B266388, Biolegend 1/200
anti-mouse CD62L PECY7, Clone MEL-14, Cat 104418, Lot B269976, Biolegend 1/200
anti-mouse CD44, Clone IM7, Cat 103027, Biolegend 1/200
anti-mouse CD44 APC, Clone IM7, Cat 563058, Lot B265921, BD 1/200
anti-mouse CD44 APC, Clone IM7, Cat 563058, Lot B265921, BD 1/200
Biolegend anti-CD44 Fitc, Clone IM7, Cat 11-0441-82, eBioscience 1/200
anti-mouse/human CD44 APC/Cyyanine 7, Cat 103027, Lot B352758 1/200
anti-mouse/human CD44 Brilliant Violet 421m, Clone IM7, Cat 103040, Lot B273304, 1/200
anti-mouse CD185 (CXCR5) bv 421, Cat 145512, Lot B357018, Biolegend 1/200
Biotin Rat anti-mouse CD185 (CXCR5), Cat 145510, Lot B21465, BD 1/200
anti-mouse PDl- BV711, Clone 29F.1Al2, Cat 135231, Lot B298663, Biolegend 1/200
anti-mouse PDl PE, Clone J43, Cat 551892, Lot 7086579, BD Bioscience 1/200
anti-mouse CD279 APC, Clone 29F.1Al2, Cat 109112, Lot B248540, Biolegend 1/200
anti mouse VB 5.1 T cell receptor Clone: MR9-4 Cat 553190 1/200
anti mouse VB 5.1 T cell receptor APC Clone: MR9-4 Cat 139506 Lot B3163556 1/200
anti-mouse CD45.l PE/Cyanine7, Clone A20, Cat 110729, Biolegend 1/200
anti-mouse CD45.1 BV421M, clone A20, Lot B376745, Biolegend. 1/200
anti-mouse CD45.1 FITC, Clone A20, cat 35-0453-U025, Lot C0453120821352, TONBO bioscience 1/200
anti-CD45.2 Mouse Monoclonal Antibody PE, Clone 104, Cat 109808, Lot B271929, 1/200
anti-mouse CD45.2, Clone 104, Cat 109808, Lot B271929, eBioscience 1/200
anti-mouse CD45.2, BV421, Clone 104, Cat 109832, Lot B357158, Biolegend 1/200
anti-mouse CD45.2 APC-Cyanine, clone 104, Cat 109824, Lot B335012, Biolegend 1/200
anti-mouse CD45.2, APC Clone 104, Cat 109814, Lot B338570, Biolegend 1/200
anti mouse CD45R/B220, Cat 563793, Lot 3135095, BD 1/200
anti-mouse CD38, Clone 90/CD38, Cat 553764, BD Bioscience 1/200
anti-mouse CD38, Clone 90/CD38, Cat 102719, Lot B371397, Biolegend 1/200
anti-mouse GL7 PB, Clone GL7, Cat 144614, Lot B306510, Biolegend 1/200
anti-mouse GL7 FITC, Clone GL7, Cat 144603, Biolegend 1/200
anti-mouse Pecy7 CD95, Clone Jo2, Cat 557653, lot 2145378, BD 1/200
anti-NK-1.1 Mouse Monoclonal Antibody PE, Clone PK136, Cat 557391, Lot 65616, 1/200
anti-mouse/human PE CD45R/B220 Antibody, Clone RA3-6B2, Cat 103208, Biolegend 1/200
anti-mouse CD86 APC, Clone GL-1, Cat 4332810, Biolegend 1/200

TotalSeqm-C0301 anti-mouse Hashtag 1 Antibody 155861, Biolegend 1ug
TotalSeqm-C0302 anti-mouse Hashtag 2 Antibody 155863, Biolegend 1ug
TotalSeqm-c0303 anti-mouse Hashtag 3 Antibody 155865,Biolegend 1ug
TotalSeqm-co304 anti-mouse Hashtag 4 Antibody 155867,Biolegend 1ug
TotalSeqm-c0305 anti-mouse Hashtag 5 Antibody, Biolegend 1ug
TotalSeqm-C0306 anti-mouse Hashtag 6 Antibody , Biolegend 1ug
TotalSeqm-C0307 anti-mouse Hashtag 7 Antibody, Biolegend 1ug
TotalSeqm-C0308 anti-mouse Hashtag 8 Antibody ,Biolegend 1ug
TotalSeqm-co309 anti-mouse Hashtag 9 Antibody,Biolegend 1ug
TotalSeqm-C0310 anti-mouse Hashtag 10 Antibody, Biolegend 1ug

Validation

All fluorescent antibodies validated on the manufacturers website.

Animals and other research organisms

Policy information about studies involving animals; ARRIVE guidelines recommended for reporting animal research, and Sex and Gender in Research

Laboratory animals

Mus musculus

	C57BL/6, SellCreERT2 ROSAtdT, TCRBKO, B6 Ightm1Mnz/J (B18hi), Tcrbtm1Mom/J, Cxcr5IRES-LoxP-STOP-LoxP-DTR FoxP3IRES-CreYFP (TFR-DTR mice)<br><br>Mice of both sexes, 6-10 Weeks of age.
Wild animals	No wild animals
Reporting on sex	sex was not considered in the study design and both sexes where therefore used.
Field-collected samples	non
Ethics oversight	All procedures in mice were performed in accordance to protocols approved by the Rockfeller University IACUC. All animal experiments were performed according to the protocols approved by the Institutional Animal Care and Use Commitee of NIAID, NIH.

Note that full information on the approval of the study protocol must also be provided in the manuscript.

Flow Cytometry

Plots

Confirm that:

- [x] The axis labels state the marker and fluorochrome used (e.g. CD4-FITC).
- [x] The axis scales are clearly visible. Include numbers along axes only for bottom left plot of group (a 'group' is an analysis of identical markers).
- [x] All plots are contour plots with outliers or pseudocolor plots.
- [x] A numerical value for number of cells or percentage (with statistics) is provided.

Methodology

Sample preparation	Single cell suspensions were obtained from popiteal lymph nodes or spleens of experimental mice, T cells and B cells were isolated by negative selection using PE-Easy Sep selection. Otherwise untouched single cell suspensions were stained for analysis.
Instrument	BD FACSSYMPHONY
Software	FACSDIVA version 8.0.2, FlowJo version v10.4.2
Cell population abundance	purity was above 95%
Gating strategy	Responsing Tfh T cells were isolated by gating of live single cells that were CD4 positive, CD62 low, CD44 high, PD1 high, CXCR5 high, and tdT + or tdT-.

- [x] Tick this box to confirm that a figure exemplifying the gating strategy is provided in the Supplementary Information.
